# Synthesis and Properties of Carbon Nanotube-Grafted Silica Nanoarchitecture-Reinforced Poly(Lactic Acid)

**DOI:** 10.3390/ma10070829

**Published:** 2017-07-19

**Authors:** Yao-Wen Hsu, Chia-Ching Wu, Song-Mao Wu, Chean-Cheng Su

**Affiliations:** 1 Department of Chemical and Materials Engineering, National University of Kaohsiung, No. 700, Kaohsiung University Road, Nan-Tzu Dist., Kaohsiung 811, Taiwan; rainwind830@gmail.com; 2 Department of Electronic Engineering, Kao Yuan University, Kaohsiung 821, Taiwan; 9113718@gmail.com; 3 Department of Electrical Engineering, National University of Kaohsiung, No. 700, Kaohsiung University Road, Nan-Tzu Dist., Kaohsiung 811, Taiwan; sungmao@nuk.edu.tw

**Keywords:** nanoarchitecture, poly(lactic acid) (PLA), nanocomposite, multi-walled carbon nanotube, silica, nanohybrid

## Abstract

A novel nanoarchitecture-reinforced poly(lactic acid) (PLA) nanocomposite was prepared using multi-walled carbon nanotube (MWCNT)-grafted silica nanohybrids as reinforcements. MWCNT-grafted silica nanohybrids were synthesized by the generation of silica nanoparticles on the MWCNT surface through the sol-gel technique. This synthetic method involves organo-modified MWCNTs that are dispersed in tetrahydrofuran, which incorporates tetraethoxysilane that undergoes an ultrasonic sol-gel process. Gelation yielded highly dispersed silica on the organo-modified MWCNTs. The structure and properties of the nanohybrids were established using ^29^Si nuclear magnetic resonance, Raman spectroscopy, wide-angle X-ray diffraction, thermogravimetric analysis, and transmission electron microscopy. The resulting MWCNT nanoarchitectures were covalently assembled into silica nanoparticles, which exhibited specific and controllable morphologies and were used to reinforce biodegradable PLA. The tensile strength and the heat deflection temperature (HDT) of the PLA/MWCNT-grafted silica nanocomposites increased when the MWCNT-grafted silica was applied to the PLA matrix; by contrast, the surface resistivity of the PLA/MWCNT-grafted silica nanocomposites appeared to decline as the amount of MWCNT-grafted silica in the PLA matrix increased. Overall, the reinforcement of PLA using MWCNT-grafted silica nanoarchitectures was efficient and improved its mechanical properties, heat resistance, and electrical resistivity.

## 1. Introduction

Carbon nanotubes (CNTs) are one of the most important nanoscale developments. The sp^2^ bonding in graphene is even stronger than the sp^3^ bonding in diamond, and it gives CNTs substantial mechanical strength. Additionally, the electronic band structure and small size of CNTs are responsible for their unique electrical, mechanical, and thermal properties [1,2,3,4]. Due to the combination of their electrical, thermal, and mechanical properties, research interest in the potential applications of CNTs in composites, electronics, computers, hydrogen storage, medicine efficiency, sensors, and other technologies and tools has grown rapidly [3,4]. Recently, nanohybrids that contain both CNTs and functional particles have attracted particular interest because of the development of CNT chemical modification methods and the search for novel functional materials [5,6,7].

An understanding of how the nanohybrid structure influences the electrical, thermal, and mechanical properties of functional particles and CNTs is critical. New nanohybrids that contain CNTs and CdSe quantum dots (QDs) have been developed using an electrostatic self-assembly method. Specifically, CdSe QDs are coated with various mercaptocarboxylic acids, after which they are self-assembled onto acridine-orange-modified CNTs via an electrostatic interaction to yield CdSe/CNT nanohybrids. The attachment of QDs onto conductive CNTs forms a metallic wire that is in direct chemical contact with the surfaces of the QDs. These metallic CNTs can then promote direct charge transport and efficient charge transfer from the QDs [8]. Gold-carbon nanotube (Au-CNT) nanohybrids combine the excellent physical and chemical properties of both gold nanoparticles and carbon nanotubes. The easily modified surfaces of gold nanoparticles and the excellent conductivity of carbon nanotubes as well as their high surface areas point toward a broad range of applications, such as biosensing, gas sensing, and electrochemistry. Zhang et al. [9] reported that surface-linked Au-CNT nanohybrids may further be classified as covalently linked and non-covalently linked. Covalently linked Au-CNT nanohybrids have only one molecule or group between a carbon nanotube and a gold nanoparticle, and the molecule is covalently bonded with a carbon nanotube and has an Au-S bond to the gold nanoparticle. Other types are known as non-covalently linked Au-CNT nanohybrids. Kim et al. [10,11] conducted a study wherein they coated CNTs with silica and synthesized mesoporous CNT-SiO_2_ nanocomposites in a two-step procedure. These mesoporous silica materials have several unique features, including a stable mesoporous structure, a large surface area, a tunable pore size and volume, and well-defined surface properties for site-specific delivery and the hosting of molecules with various sizes, shapes, and functionalities. Mesoporous CNT-SiO_2_ composites have performed well in the size-selective adsorption of biomacromolecules, thus exhibiting great potential for use in biomacromolecular separation [12,13].

Recently, many methods of functionalizing carbon nanotubes with covalent bonds have been investigated [9,14,15,16,17,18,19]. Chen et al. [14] gave the first description of covalently functionalized single walled carbon nanotubes. Oxidized carbon nanotubes with carboxylate groups can be used to bond with –OH and –NH_2_, while crude carbon nanotubes can be directly functionalized with R–(CO)–O–O–(CO)–R. These covalent bonding methods have been demonstrated for nanoparticle deposition on carbon nanotubes [15]. Marsh et al. [16] modified multi-walled carbon nanotubes with 2-aminoethanethiol and assembled gold nanoparticles on these carbon nanotubes by bonding via the thiolate group. Coleman et al. [17] covalently deposited gold nanoparticles on single walled carbon nanotubes. This deposition is based on the reaction of carboxylate groups on carbon nanotubes with amino groups in 2-aminoethanethiol and the reaction of the thiol group in 2-aminoethanethiol with gold nanoparticles. Zanella et al. [18] investigated the deposition of gold nanoparticles on different aliphatic bifunctional thiol-modified carbon nanotubes. Also, based on the carboxylate group on the carbon nanotubes, HSCH_2_CH_2_OH can be modified on carbon nanotubes through the reaction of –OH and –COOH groups in order to immobilize gold nanoparticles. Moreover, using the reaction of the aryl group with carbon nanotubes, one can covalently deposit gold nanoparticles on carbon nanotubes [19].

CNT-inorganic nanoparticle hybrids that are prepared using the sol-gel method have rapidly become a widespread target of research in materials science. Sol-gel procedures that are used to fabricate metal oxides begin from a chemical solution that acts as a precursor in the formation of an integrated network of either discrete particles or network polymers. Typical precursors are metal alkoxides and metal chlorides, which undergo various hydrolysis and polycondensation reactions. Polymerization is associated with the formation of a one-, two-, or three-dimensional network of metal-oxide (M–O–M) bonds and is accompanied by the production of H–O–H and R–O–H species [20,21,22]. The influence of synthetic conditions, such as the applied metal alkoxide concentration, pH, temperature, catalyst morphology, and inorganic metal oxide structure, has been thoroughly studied in recent decades [20,21,22,23,24,25,26]. In our own earlier research [7], we examined tetraisopropyl titanate (TIPT), a metal alkoxide that underwent various hydrolysis and polycondensation reactions and formed a network of titanium oxide bonds. Additionally, functionalized multi-walled CNTs (MWCNTs) with organic functionalized groups have been used to form CNT-grafted titania nanohybrids with a network structure of titania between the CNTs [7,21,22].

Recently, developing biodegradable polymers with excellent properties has become a key challenge for materials science, with one renewable polymer, poly(lactic acid) (PLA), being the most promising candidate in these endeavors. In fact, PLA that is derived from renewable resources (e.g., starch) is now regarded as a promising alternative to the traditional petrochemical polymers because it can address concerns regarding environmental pollution, greenhouse gas emissions, and fossil resource depletion [27,28,29]. PLA is also a thoroughly studied environmentally friendly polymer because of its favorable strength and stiffness, which allow it to have a wide range of applications in biomedical materials, electronics, packaging materials, degradable plastic bags and bottles, and cars. Thus, PLA has a notable commercial potential for bioplastic applications, although some of its properties, including brittleness, low heat deflection temperature (HDT), and low melt viscosity for processing, have restricted its widespread use to date. Therefore, the modification of renewable PLA through innovative technology is a necessary task for materials scientists. Nanoreinforcements of PLA are promising with respect to the design and development of green nanocomposites for many applications. The new nanocomposites are also significant because of their nanoscale dispersion, which results in high aspect ratios and large surface areas [29].

In the present study, a novel nanocomposite combining biodegradable PLA and functional nanohybrids, which formed eco-friendly composites, was developed. The addition of nanofillers is a unique strategy to extend and improve the properties of PLA for various end uses; in this study, we performed the covalent modification of surface-functionalized MWCNTs to synthesize MWCNT-grafted silica nanoparticles. Adding silica particles to MWCNTs was found to support the development of electrical devices or structures by exploiting the favorable physical characteristics of MWCNTs. Additionally, silica-modified MWCNTs could be regarded as ideal nanofillers in the formation of PLA-based nanocomposites because they improved the mechanical properties, heat resistance, and electrical conductivity.

## 2. Experimental

### 2.1. Materials and Methods

PLA was purchased from SUPLA Material Technology Ltd. (SUPLA 135N, M_n_ = 52,000 g/mol, Tainan, Taiwan) and the MWCNTs (Baytubes C150 HP) were purchased from BASF (Ludwigshafen, Germany). The development of functionalized MWCNTs using carboxylic acid, acyl chloride, amine, and hydroxyl groups was reported in our previous study [7]. Treating MWCNTs with carboxylic acid modifies their surface through the covalent attachment of organic acid to their π-conjugated skeletons. MWCNTs (0.1 g), sulfuric acid (8 mL), and nitric acid (24 mL) were placed in a 125 mL round-bottom flask that was equipped with a condenser and stirrer. The chemical oxidation reaction was performed using an ultrasonic apparatus at 60 °C for 48 h. Following acid treatment, the MWCNTs were functionalized with carboxylic acid on their surfaces. Next, another 0.1 g of MWCNTs was placed in a 250 mL flat-bottom flask with 100 mL of ethylene chloride. The mixture was sonicated for 1 h to disperse the MWCNTs in the solvent. Subsequently, 15 mL of thionyl chloride was added to the flat-bottom flask and the mixture was stirred using a magnetic stirrer and refluxed for 24 h. Next, the unreacted thionyl chloride and solvent were distilled and 50 mL of allyl alcohol was stirred into the acid chloride MWCNTs at 50 °C for 24 h. The synthesized acyl-chloride-functionalized MWCNTs were then washed in acetone to remove unreacted chemicals, and they were reacted with ethylene glycol at 80 °C for 12 h. Meanwhile, hydroxyl group-functionalized MWCNTs were synthesized, and the products were washed with ethanol to remove unreacted chemicals. Later, the acyl-chloride-functionalized MWCNTs were reacted with hexamethylene diamine in tetrahydrofuran (THF) at 50 °C for 12 h; similarly, amine-functionalized MWCNTs were synthesized, and the products were washed with THF to remove unreacted chemicals. Finally, the surface-functionalized MWCNTs were dried in a vacuum oven at 50 °C for 12 h [6,7].

**Scheme 1:**
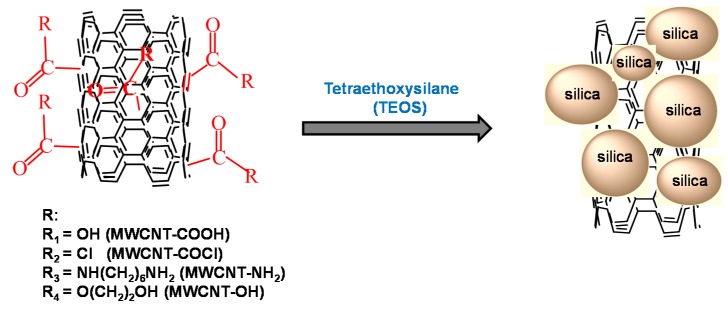
Synthesis of multi-walled carbon nanotube (MWCNT)-grafted silica nanohybrids.

The MWCNTs were initially functionalized with carboxylic acid, acyl chloride, amine, and hydroxyl groups, and they were then dispersed in a tetraethoxysilane (TEOS) solution through ultrasonic processing at room temperature; neat silica was similarly synthesized. Following gelation, well-dispersed silica in the MWCNT-grafted silica nanohybrids was obtained. Scheme 1 represents the synthesis of MWCNT-grafted silica nanohybrids.

In the study, the MWCNTs that were functionalized with carboxylic acid formed MWCNT-grafted silica nanoarchitectures; this fostered the development of MWCNT-grafted silica-reinforced PLA nanocomposites. PLA/MWCNT-grafted silica and PLA/MWCNT nanocomposites were prepared by a melt-compounding process using a Haake twin-screw extruder. The temperature of the heated zone from the hopper to the die was set to 180 °C, 190 °C, 195 °C, and 185 °C, and the rotation was set to a constant speed of 30 rpm. To fabricate PLA/MWCNT-grafted silica nanocomposites, PLA was melt-compounded with the addition of MWCNT-grafted silica at 0.05 and 0.5 wt % in the polymer matrix; PLA/MWCNT nanocomposites were similarly prepared.

### 2.2. Characterization

Thermogravimetric analysis (TGA) of the surface-functionalized MWCNTs was conducted using a thermogravimetric analyzer (TGA2950, TA Instruments Inc., New Castle, DE, USA). The samples were evenly and loosely distributed in an open sample pan of 6.4 mm diameter and 3.2 mm depth with an initial sample weight of 6–10 mg. The temperature change was controlled from 75 °C to 650 °C at a heating rate of 5 °C/min. High purity nitrogen was continuously passed into the furnace at a flow rate of 80 mL/min. A Fourier transform infrared spectrometer (FTIR; PerkinElmer Spectrum 100, PerkinElmer Inc., Waltham, MA, USA) was used to identify the functional groups of the MWCNT-grafted silica nanohybrids. Spectra were obtained with a resolution of 4 cm^−1^, and their averages were obtained from at least 64 scans within the standard wavenumber range of 500–4000 cm^−1^. The characterization of MWCNT-grafted silica nanohybrids was achieved by Raman spectroscopy using the 532 nm line of a YAG laser, which was operated at a laser power of 500 mW. A laser with a beam diameter of approximately 2 μm was focused with a 1000× objective onto the surface of the MWCNTs. Solid-state ^29^Si NMR measurements were recorded at room temperature using a Bruker Avance 400 NMR spectrometer (Bruker Corp., Billerica, MA, USA) operating at 400 MHz for ^29^Si. Solid ^29^Si cross-polarization and magic-angle spinning NMR experiments were performed using a Bruker MSL-400 spectrometer operating at 400 MHz. For wide-angle X-ray diffraction (WAXD, Shimadzu Corp., Kyoto, Japan) experiments, a Shimadzu/XRD-6000 instrument with copper K_α_ radiation (λ = 0.1542 nm) was used. The scanning 2*θ* angle covered a range between 5 and 55° in steps of 0.2°. The microstructure of the MWCNT-grafted silica nanohybrids was analyzed by transmission electron microscopy (TEM, model JEOL JEM 1200 EX, JEOL Ltd., Tokyo, Japan) at an acceleration voltage of 100 kV. Tensile tests on the composites were carried out using a computerized universal testing machine (Instron 4465, Instron Engineering Corp., Norwood, MA, USA) according to the ASTM D-638 standard at a pull up speed of 5 mm/min at 25 °C. Five specimens were tested for each condition to minimize errors. The yield strength was estimated based on their stress-strain curve. The HDT of the PLA/MWCNT-grafted silica nanocomposite was measured with a Cotech HV-3000-PE instrument (Cotech Inc., Taipei, Taiwan) according to ASTM D-648 under a load of 0.455 MPa at a uniform heating rate of 2 °C/min. The temperature at which a test bar loaded to the specified bending stress deflects by 0.25 mm was noted as the HDT. The surface resistivity of the nanosamples was measured according to ASTM D-257 using a Keithley 6517A electrometer (Keithley Instruments Inc., Solon, OH, USA). The 3 mm thick and 75 mm diameter specimens were copper-plated for better contact after subjecting them to humidity conditioning at 95% RH and 35 °C.

## 3. Results and Discussion

### 3.1. Analysis of Surface-Functionalized MWCNTs

**Figure 1:**
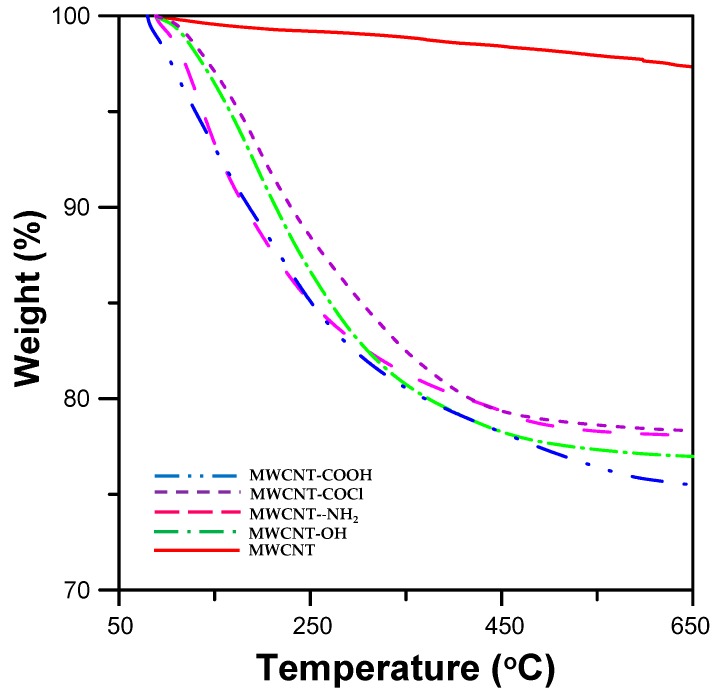
Thermal degradation of surface-functionalized MWCNTs.

The thermal degradation of organic material involves molecular deterioration as a result of overheating. The components of the chain backbone of the organic material begin to break (chain scission) and then gasification occurs at high temperatures [30,31]. In this study, the degree of surface functionalization of MWCNTs with various surface modifiers was estimated using TGA. Before the start of each run, nitrogen was used to purge the furnace for 30 min to establish an inert environment in order to prevent any unwanted oxidative decomposition. Figure 1 shows the thermal stability of surface-functionalized MWCNTs; notably, no appreciable loss of MWCNT mass occurred during the heating procedure. Indeed, the 18.16–20.02% mass loss was due to organic group gasification. In particular, surface-functionalized MWCNTs clearly exhibit a loss of mass, while an organic modifier on the surface of the MWCNTs can be gasified in flowing nitrogen. TGA revealed that the degree of surface functionalization of the MWCNTs depended on this loss of mass. The degree of surface functionalization was calculated from the calcination of the MWCNTs using the following equation:

(1) $$\frac{B-A}{M}=C$$

where *A* is the mass loss of the crude MWCNTs (%), *B* is the mass loss of the surface-functionalized MWCNTs (%), *M* is the molecular weight of the functional group (g/mol), and *C* is the degree of surface functionalization of the MWCNTs (mol %). The mol % values of the carboxylic acid, acyl chloride, hydroxyl group, and amine used to functionalize the surface of the MWCNTs ranged from 0.11 to 0.37 mol %. In particular, the value for carboxylic acid-modified MWCNTs was 0.37 mol %. The degree of surface functionalization of the MWCNTs is presented in Table 1.

### 3.2. Characterization of MWCNT-Grafted Silica Nanohybrids

**Figure 2:**
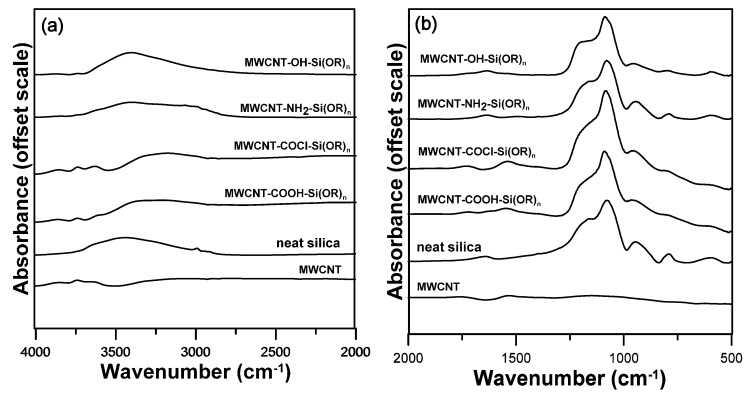
FT-IR spectra of MWCNT-grafted silica nanohybrids: (**a**) the IR region between 2000 and 4000 cm^−^^1^ and (**b**) the IR region between 500 and 2000 cm^−1^.

Various functional groups were synthesized in this study to functionalize MWCNTs. Carboxylic acid-, acyl chloride-, hydroxyl group-, and amine-modified MWCNTs interacted with hydrophilic silica to form MWCNT-silica nanohybrids. Infrared (IR) spectra of the MWCNT-grafted silica nanohybrids are shown in Figure 2, revealing the chemical interaction between functionalized MWCNTs and the TEOS solution. Figure 2a shows the IR absorption in the wavenumber range 2000–4000 cm^−1^. The characteristic absorption peak of the hydroxyl group was observed in the 3000–3700 cm^−1^ range. Figure 2b shows the IR absorption in the wavenumber range 500–2000 cm^−1^. The characteristic absorption peaks of the silicon oxide group were observed in the 1000–1300 cm^−1^ range. These results are similar to those observed in our previous study [7], which also examined surface-functionalized MWCNTs and demonstrated that the main characteristic absorption peak of the hydroxyl group was in the 3200–3700 cm^−1^ range of the IR spectra. Additionally, in the present study, the absorption peaks of the amine, carboxylic acid, and acyl chloride groups were observed at 3000, 3300, and 800 cm^−1^, respectively. The MWCNTs were initially functionalized with the carboxylic acid, acyl chloride, amine, and hydroxyl groups and were then dispersed in a TEOS solution by ultrasonic processing at room temperature. In the MWCNT-grafted silica nanohybrids, the sol-gel process reduced the IR absorption intensities of the four functional groups on the surface of the MWCNTs; the silicon oxide group was simultaneously observed in the MWCNT-grafted silica nanohybrids. Therefore, more polar functional groups were attached to the surface of chemically modified MWCNTs, and MWCNT-grafted silica nanohybrids were yielded.

**Figure 3:**
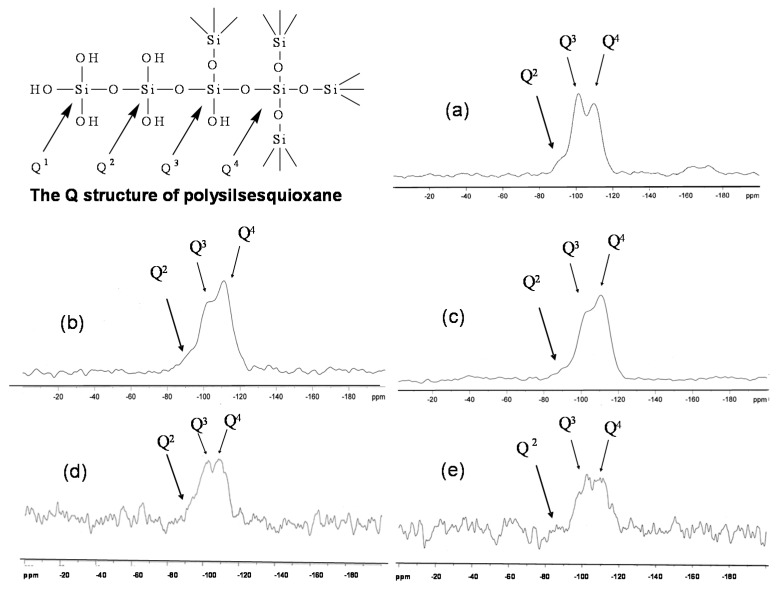
The solid-state ^29^Si NMR spectra of silica and MWCNT-grafted silica nanohybrids: (**a**) neat silica; (**b**) MWCNT-COOH-silica; (**c**) MWCNT-COCl-silica; (**d**) MWCNT-NH_2_-silica; and (**e**) MWCNT-OH-silica.

As noted, MWCNT-grafted silica nanohybrids were synthesized by the generation of silica nanoparticles on the surface of MWCNTs using the sol-gel technique. Specifically, siloxane alkoxide underwent various hydrolysis and polycondensation reactions, which formed a network of siloxane bonds in the MWCNT-grafted silica nanohybrids [20]. When the nanohybrids were in a solid-state, ^29^Si NMR spectra of the network of siloxane bonds showed three signals at −92, −101, and −111 ppm, which were assigned to (HO)_2_Si(SiO)_2_ (Q^2^), HOSi(SiO)_3_ (Q^3^), and SiO_4_ (Q^4^), respectively [20,26,32,33]. Figure 3 shows the solid-state ^29^Si NMR spectra of MWCNT-grafted silica nanohybrids as well as those of neat silica, revealing their structure and properties and the Q architecture of the polysilsesquioxane. Notably, the strong signals at −101 and −111 ppm suggest that the formation of the silica network in the prepared samples was complete; in other words, the siloxane in the MWCNT-grafted silica nanohybrids was completely reacted. However, the proportion of the formed Q^2^ that was observed in the neat silica indicated that the siloxane had only partially reacted in the neat silica network structure.

TEOS is the ideal chemical precursor in sol-gel synthesis because it reacts readily with water. The mechanism of the sol-gel process is as follows [20,26,32,33,34,35]. 

Hydrolysis:

Si(OR)_4_ + H_2_O → HO–Si(OR)_3_ + R–OH (2)

Condensation:

(OR)_3_–Si–OH + HO–Si–(OR)_3_ → [(OR)_3_Si–O–Si(OR)_3_] + H–O–H (3)

**Scheme 2:**
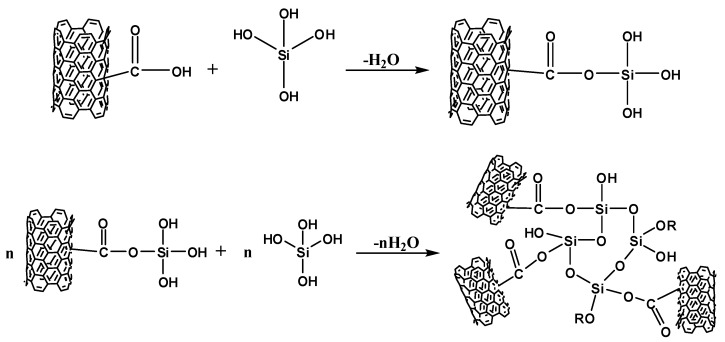
The mechanism of formation of carboxylic acid-modified MWCNT-grafted silica nanohybrids.

In sol-gel synthesis, polymerization first yields a one-, two-, or three-dimensional network of siloxane (Si–O–Si) bonds, accompanied by the formation of H–O–H and R–O–H species. Then, a network structure of silica among the MWCNTs is developed in MWCNT-grafted silica nanohybrids. Scheme 2 provides an example of this process, showing the mechanism for developing carboxylic acid-modified MWCNT-grafted silica nanohybrids.

**Figure 4:**
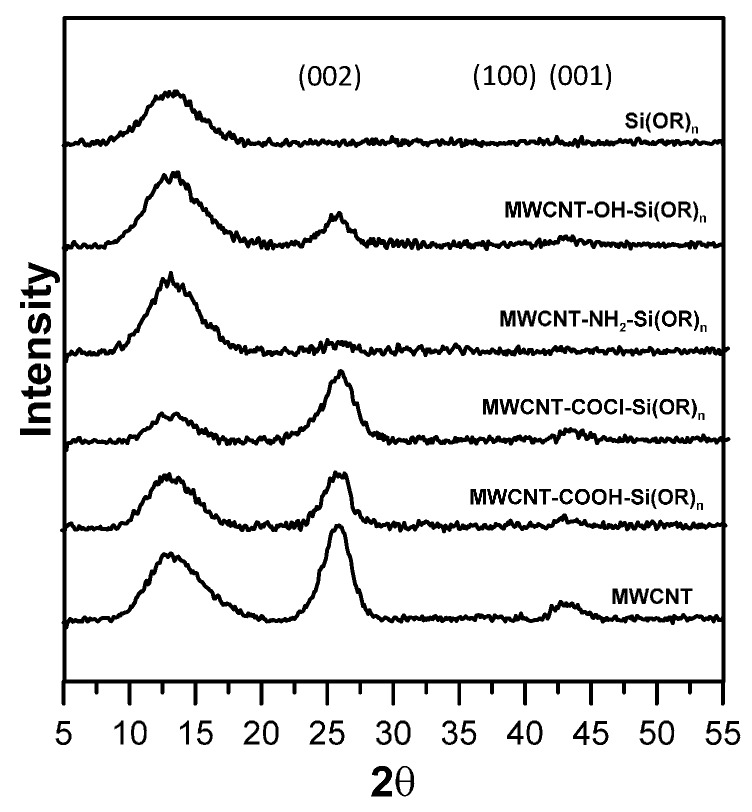
XRD patterns of MWCNT-grafted silica nanohybrids.

In Figure 4, the WAXD patterns of MWCNT-grafted silica nanohybrids, as well as those of neat MWCNTs and silica, are presented. The peaks of the MWCNTs, indexed to (002), (100), and (101), reflect their crystalline hexagonal structure; in particular, the (002) peak suggests that the MWCNTs are multi-walled [36]. Additionally, the neat silica WAXD patterns in the range 2*θ* = 10°–17° show that the neat silica has an amorphous structure. Similarly, the MWCNT-grafted silica nanohybrid WAXD patterns reveal that silica is only in the amorphous phase. The WAXD pattern of the surface-functionalized MWCNTs was observed to be very similar to that of the neat MWCNTs, with both patterns including one strong peak and two weak peaks. Therefore, the crystalline structure of the activated MWCNTs was considered to be the same as that of the neat MWCNTs; in other words, the functionalization process did not damage the surface structure of the neat MWCNTs. We concluded that the amorphous phase demonstrated by WAXD patterns between 2*θ* = 10° and 17° might be a result of both the MWCNT and silica amorphous phases.

**Figure 5:**
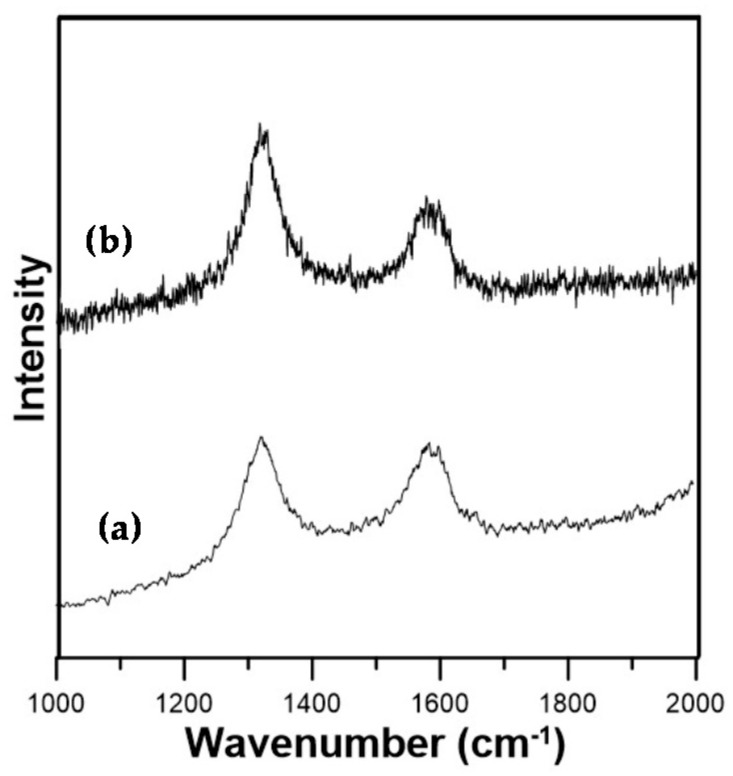
Raman spectra of MWCNTs: (**a**) crude MWCNT; (**b**) carboxylic acid-modified MWCNT-grafted silica nanohybrids.

Raman spectroscopy is a versatile, nondestructive method for identifying multiple structural phases that may coexist in a sample [6,7,37]. In this study, Raman spectroscopy was used to detect amorphous and crystalline phases in MWCNT samples with various degrees of graphitization. Figure 5 shows the Raman spectra of the crude MWCNTs and carboxylic acid-modified MWCNT-grafted silica nanohybrids. The characteristic peaks of the MWCNTs at 1326 cm^−1^ (D-band) and 1580 cm^−1^ (G-band) were attributed to amorphous carbon and well-graphitized MWCNTs, respectively. Notably, the broad peak at 1326 cm^−1^ may indicate the presence of a defective graphitic layer or carbon particles that remained even after the purification procedure. Moreover, the ratio of the intensity of the D-band peak to that of the G-band peak (ID/IG) of the crude MWCNTs was 1.35, whereas that for the MWCNT-grafted silica nanohybrids was 1.97. This reveals that more defective graphitic layers were formed by the acid treatment. Finally, the chemical bonding of MWCNTs in the nanohybrids preserved the nanotube structure after grafting, which was evidenced by the fact that the relative intensity of the Raman G-band of MWCNT-grafted silica nanohybrids was moderately lower than that of the neat sample [7,37].

### 3.3. Morphology of CNT-Grafted Silica Nanohybrids

The homogeneous distribution and dispersion of fillers within a host matrix are common prerequisites for the formation of nanohybrids. In addition, the dispersion must be stabilized to prevent filler agglomeration. Mixing through the sol-gel method disperses silica, while chemical modification helps to stabilize the dispersion and allows silica to engage with the CNTs. Therefore, surface-functionalized MWCNTs are attractive because their functionalization may improve the interaction between the MWCNTs and silica.

**Figure 6:**
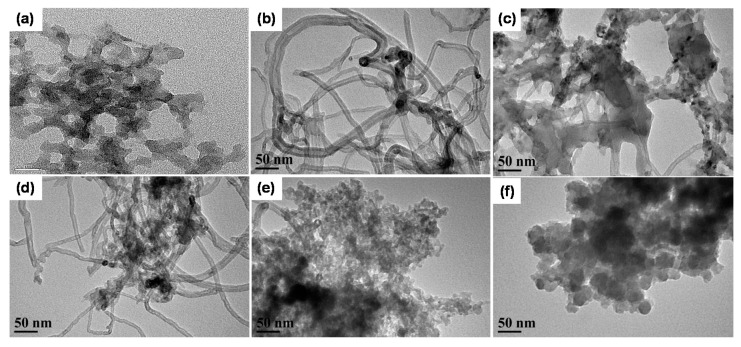
TEM images of MWCNT-grafted silica nanohybrids: (**a**) neat silica (Si(OR)_n_); (**b**) crude MWCNTs; (**c**) MWCNT-COOH-silica; (**d**) MWCNT-COCl-silica; (**e**) MWCNT-NH_2_-silica; and (**f**) MWCNT-OH-silica.

Following the sol-gel process, which involved a TEOS-to-MWCNT weight ratio of 2:1 and was performed at 25 °C for 24 h, the morphologies of the organo-modified MWCNT-grafted silica were observed using TEM. The images are shown in Figure 6. Figure 6a depicts an image of the neat silica nanoparticles (Si(OR)_n_) that were synthesized using nitric acid as a catalyst, revealing the existence of a coral-like crosslinked network in the silica. Figure 6b shows an image of the crude MWCNTs. The length of the MWCNTs was several tens of microns and their diameter was approximately 10–30 nm. Figure 6c–f shows TEM micrographs of the MWCNT-COOH-silica, MWCNT-COCl-silica, MWCNT-NH_2_-silica, and MWCNT-OH-silica nanohybrids, respectively. The morphology and structure of the formed colloidal silica particles depended on the molar ratio of the reagents. For example, hydrolyzed TEOS self-condenses to form large continuous silica networks with mesoporous structures, especially in the presence of a high or excessive concentration of the TEOS reagent [20]. In an earlier study [7], the aggregation of chemically modified MWCNTs was determined to be less than that of crude MWCNTs because the carboxylic acid, acyl chloride, amine, and hydroxyl functional groups on the surface of the modified MWCNTs (as well as the short length and low aspect ratio of the modified MWCNTs) increased their polarity. Thus, the functionalization of MWCNTs with carboxylic acid, acyl chloride, amine, and hydroxyl groups during the sol-gel process yielded MWCNT-grafted silica nanohybrids with a network structure of silica among the MWCNTs.

MWCNTs were incorporated into silica particles, not only to prevent the agglomeration of nanofillers but also to increase the surface area of the nanohybrids. Consequently, the MWCNT-grafted silica nanohybrids in our experiment featured relatively homogeneous silica without obvious agglomeration. By mapping the distribution of silicon, we concluded that the inorganic silica nanoparticles in the MWCNT-grafted silica nanohybrids were distributed uniformly on the nanometer scale and that their nanoarchitectures were well-defined.

### 3.4. PLA/MWCNT-Grafted Silica Nanocomposites

**Figure 7:**
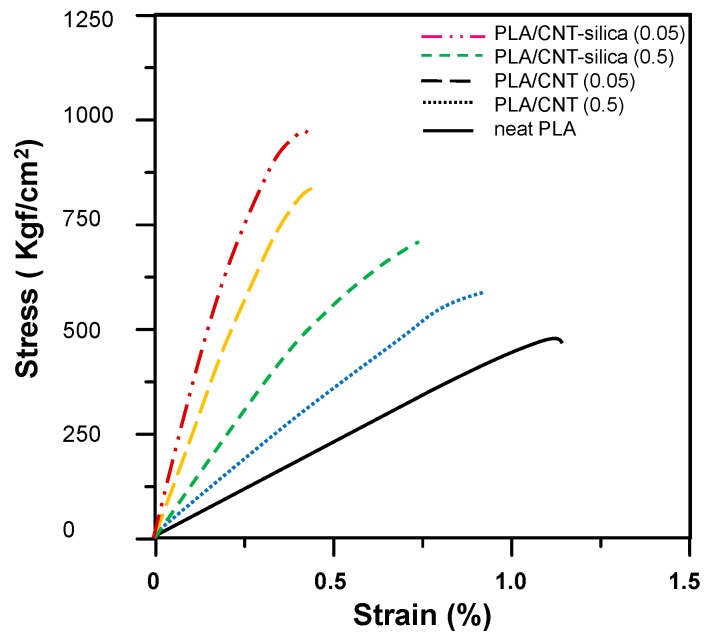
Stress–strain curves of neat poly(lactic acid) (PLA) and PLA-based nanocomposites.

PLA suffers from various shortcomings, such as a low thermal resistance and a low rate of crystallization, which limit its range of application in many fields. Adding nanofillers is an effective means of improving the properties of PLA for use in PLA composites [29]. In the present study, the MWCNTs that were functionalized with carboxylic acid formed MWCNT-grafted silica (MWCNT-CO–OH-silica) nanoarchitectures; this fostered the development of MWCNT-grafted silica-reinforced PLA nanocomposites, which were prepared using a melt-compounding process in a twin-screw extruder. Figure 7 shows the stress-strain curves and variation in the mechanical properties of the PLA-based nanocomposites containing MWCNT or MWCNT-grafted silica. Notably, the tensile strength of the PLA/MWCNT and PLA/MWCNT-silica nanocomposites increased as the MWCNT or MWCNT-silica content increased because of the nano-reinforcing effects of MWCNT and MWCNT-silica. The MWCNT-grafted silica nanoarchitecture, comprising many silica nanoparticles on the surface of its MWCNTs, had a higher aspect ratio than did the crude MWCNT nanoarchitecture (0.05 vs. 0.5 wt % nanofiller, respectively). Thus, tensile strength can be better enhanced using PLA/MWCNT-grafted silica nanocomposites than by using PLA/MWCNT nanocomposites. The mechanical properties of the PLA/MWCNT and PLA/MWCNT-silica nanocomposites also improved substantially upon the incorporation of a small amount of MWCNT or MWCNT-grafted silica; notably, this improvement was more marked with a lower nanofiller content. Furthermore, the elongation at break gradually decreased as the MWCNT content increased, indicating that the PLA/MWCNT and PLA/MWCNT-silica nanocomposites were made more brittle by the incorporation of MWCNT and MWCNT-silica, respectively.

A list of the mechanical, electrical, and thermal properties of neat PLA, PLA/MWCNT, and PLA/MWCNT-silica nanocomposites is presented in Table 2. The incorporation of MWCNT and MWCNT-grafted silica into the PLA matrix improved the physical properties of the PLA-based nanocomposites. Specifically, for the PLA/MWCNT nanocomposites, the tensile strengths were 615.9 and 856.3 Kgf/cm^2^ for PLA with 0.05 and 0.5 wt % MWCNTs, respectively. However, for the PLA/MWCNT-silica nanocomposites, the tensile strengths were 710.3 and 975.6 Kgf/cm^2^ for PLA with 0.05 and 0.5 wt % MWCNT-silica, respectively. In other words, the PLA/MWCNT-silica nanocomposites had a more enhanced tensile strength than did the PLA/MWCNT nanocomposites. However, both the PLA/MWCNT-silica and PLA/MWCNT nanocomposites were brittle, as shown by the small elongation at break of the samples.

The surface resistivity of the PLA/MWCNT-silica nanocomposites decreased as the MWCNT-grafted silica content increased. This resulted from the nanotube-nanotube links, which became more dominant as higher amounts of MWCNT-grafted silica were added and formed interconnected structures in the PLA/MWCNT-silica nanocomposites. These interconnected structures then formed MWCNT-silica agglomerates, resulting in PLA/MWCNT-grafted silica nanocomposites with high electrical conductivity. As revealed in Table 2, the PLA/MWCNT and PLA/MWCNT-grafted silica nanocomposites exhibited much higher HDT values than did neat PLA, and these values varied dramatically with the amount of MWCNT-grafted silica or MWCNT. Specifically, the neat PLA had a low HDT (95.1 °C). For the PLA/MWCNT nanocomposites, the HDTs were 130.5 °C and 134.2 °C for PLA with 0.05 and 0.5 wt % CNT, respectively. However, the HDTs of the PLA/MWCNT-silica nanocomposites were 136.7 °C and 143.8 °C for PLA with 0.05 and 0.5 wt % CNT-silica, respectively. The higher HDTs were triggered by MWCNT-silica, which has high aspect ratios. Nevertheless, the HDTs and mechanical properties of the PLA/MWCNT and PLA/MWCNT-silica nanocomposites were also appreciably improved by the incorporation of a very small amount of MWCNTs. In the semicrystalline polymer, the HDTs and mechanical properties are not only proportional to the content of nanofillers in the composites but also increase with the extent to which the polymer is crystallized [38,39,40,41,42].

The addition of a crystalline nucleating agent to polymers can effectively promote the formation of polymer segments with an ordered structure, which can increase the rate and extent of polymer crystallization. Generally, MWCNTs can act as such an agent in nanocomposites [29,43,44,45,46]. Park et al. [29] suggested that a small amount of MWCNTs acted as a nucleating agent, improving the crystallization, mechanical properties, and thermal resistance of PLA/MWCNT composites. The present study similarly demonstrated that MWCNTs substantially reinforced PLA by acting as nucleating agents in the PLA matrix, thereby improving the physical properties of the resulting nanocomposites. In short, nanofillers, MWCNTs, and MWCNT-silica could enhance the physical properties of the PLA-based nanocomposites. However, the reinforcement of nanoarchitectures using MWCNT-grafted silica more efficiently reinforced PLA and improved its electrical, mechanical, and thermal properties.

**Figure 8:**
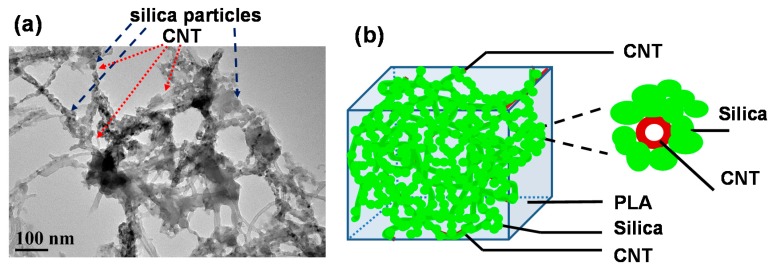
TEM microphotograph of PLA/MWCNT-silica nanocomposites: (**a**) nanocomposite with a MWCNT-silica content of 0.5 wt % and (**b**) schematic depiction of the MWCNT-grafted silica in the PLA matrix.

Figure 8 shows TEM images of PLA-based nanocomposites with 0.5 wt % MWCNT-COOH-silica, which were produced by the chemical interaction between carboxylic acid-functionalized MWCNTs and TEOS. The morphology of the PLA/MWCNT-silica nanocomposites in the image reveals a uniform dispersion of silica particles on the surface of the MWCNTs and highly entangled MWCNTs in the PLA matrix; the silica can also be clearly distinguished morphologically from the MWCNTs (i.e., the black spots in Figure 8a). Figure 8b provides a schematic depiction of the three-dimensional structure of the PLA/MWCNT-grafted silica nanocomposites, confirming that the MWCNT-grafted silica in the PLA-based nanocomposites was distributed uniformly on the nanometer scale. The nanoarchitectures of these nanocomposites were also well defined.

## 4. Conclusions

A novel PLA-based nanocomposite was prepared using MWCNT-grafted silica nanohybrids as reinforcements. The organo-modified MWCNTs can be used to produce MWCNT-grafted silica nanohybrids with widely dispersed silica. The degree of surface functionalization of the MWCNTs was examined by thermogravimetric analysis. The surface-functionalized MWCNT-grafted silica nanoarchitectures were prepared using the sol-gel technique, which helped to improve the compatibility between the MWCNTs and the inorganic silica particles. Siloxane bridges in the silica were observed and were found to be highly crosslinked in the MWCNT-grafted silica nanohybrids. MWCNT-grafted silica nanohybrids have several unique features that are not shared by traditional silica-coated MWCNTs, such as special nanoarchitectures and the ability to host molecules with various sizes, shapes, and functionalities. Moreover, MWCNTs that are functionalized with various organo-functional groups form MWCNT-grafted silica nanoarchitectures, which have specific and controllable morphologies. Additionally, PLA/MWCNT-grafted silica (MWCNT-COOH-silica) nanocomposites were studied using a melt-compounding process in a twin-screw extruder, and it was determined that their mechanical properties, surface resistivity, and heat resistance were considerably improved by the incorporation of a very small amount of MWCNT-grafted silica; these improvements were significant with lower MWCNT-silica contents. Overall, this study demonstrated that MWCNT-grafted silica can substantially reinforce PLA and improve the physical properties of the resulting nanocomposites. However, although the MWCNT and MWCNT-silica nanofillers improved the physical properties of the PLA-based nanocomposites, MWCNT-grafted silica more efficiently reinforced PLA and more effectively improved its mechanical properties, heat resistance, and electrical resistivity.

## Figures and Tables

**Table 1:** Degree of surface-functionalization of MWCNTs.

Surface-Functionalized MWCNTs	Mass Loss of Surface-Functionalized MWCNT (wt %)	Degree of Surface-Functionalization of MWCNTs (mol %)
MWCNT-COOH	19.23%	0.37
MWCNT-COCl	18.16%	0.25
MWCNT-CONH(CH_2_)_6_NH_2_	18.35%	0.11
MWCNT-COO(CH_2_)_2_OH	20.02%	0.21
MWCNT	2.50%	---

**Table 2:** Physical properties of neat PLA and PLA-based nanocomposites.

Properties	Neat PLA	PLA/MWCNT-Silica		PLA/MWCNT	
		0.05 wt %	0.5 wt %	0.05 wt %	0.5 wt %
Tensile strength (Kgf/cm^2^)	470.6	710.3	975.6	615.9	856.3
Elongation (%)	1.2	0.8	0.5	0.9	0.5
Surface resistivity (ohms/sq)	2.0 × 10^12^	2.6 × 10^7^	4.7 × 10^4^	8.8 × 10^7^	4.5 × 10^4^
HDT (°C)	95.1	134.2	143.8	130.5	136.7
